# Inhibition of Extracellular Calcium Influx Results in Enhanced IL-12 Production in LPS-Treated Murine Macrophages by Downregulation of the CaMKK**β**-AMPK-SIRT1 Signaling Pathway

**DOI:** 10.1155/2016/6152713

**Published:** 2016-05-30

**Authors:** Xin Liu, Ning Wang, Yuanfeng Zhu, Yongjun Yang, Xiaoli Chen, Shijun Fan, Qian Chen, Hong Zhou, Jiang Zheng

**Affiliations:** ^1^Medical Research Center, Southwest Hospital, The Third Military Medical University, Chongqing 400038, China; ^2^Department of Pharmacology, College of Pharmacy, The Third Military Medical University, Chongqing 400038, China

## Abstract

Activated macrophages are the primary sources of IL-12, a key cytokine bridging innate and adaptive immunity. However, macrophages produce low amounts of IL-12 upon stimulation and the underlying regulatory mechanism remains unclear. In this study, we found a new calcium-dependent mechanism that controlled IL-12 production in LPS-treated murine macrophages. First, LPS was demonstrated to induce extracellular calcium entry in murine peritoneal macrophages and inhibition of calcium influx resulted in marked enhancement in IL-12 production. Then, withdrawal of extracellular calcium was found to suppress CaMKK*β* and AMPK activation triggered by LPS while chemical inhibition or genetic knockdown of these two kinases augmented LPS induced IL-12 production. AMPK activation increased the NAD^+^/NADH ratio and activated Sirtuin 1 (SIRT1), a NAD^+^-dependent deacetylating enzyme and negative regulator of inflammation. Chemical inhibitor or siRNA of SIRT1 enhanced IL-12 release while its agonist suppressed IL-12 production. Finally, it was found that SIRT1 selectively affected the transcriptional activity of NF-*κ*B which thereby inhibited IL-12 production. Overall, our study demonstrates a new role of transmembrane calcium mobilization in immunity modulation such that inhibition of calcium influx leads to impaired activation of CaMKK*β*-AMPK-SIRT1 signaling pathway which lifts restriction on NF-*κ*B activation and results in enhanced IL-12 production.

## 1. Introduction

The recruitment and activation of macrophages in response to microbial products such as lipopolysaccharide (LPS) lead to production of various immune/inflammatory effectors, which are essential for the anti-infectious immune response. Interleukin 12 (IL-12), a 70 kD (p70) heterodimeric cytokine comprised of a p40 and a p35 subunit separately, is mainly produced by activated macrophages and plays a pivotal role in connecting innate immunity with adaptive immunity [1]. The expression of IL-12 is primarily modulated at transcriptional level in macrophages. Indeed, various control elements have been revealed in the expression of inducible p40 gene. For example, it was found that a sequence in NF-*κ*B (p50/p65 and p50/c-Rel) complexes was functionally essential for modulating the promoter activity of IL-12 p40 in response to several p40-inducing pathogens [2, 3]. Other regulatory elements, such as C/EBP, ETS-2, and IRF1, have been also implicated in mediating the inducible promoter activity of IL-12 [1, 4–6]. However, the upstream signaling events that connect microbial stimulation with downstream transcriptional regulation to control IL-12 expression in macrophages remain largely unknown.

Extracellular calcium influx across the plasma membrane is a major source for the increase of intracellular calcium, which acts as a crucial secondary messenger in regulating macrophage activation, such as phagosomes maturation, activation of NLRP3 inflammasome, and transcriptional control of cytokines [7–9]. Microbial components (LPS, etc.) are known to elicit calcium influx in murine macrophages and thereby upregulate the production of mediators like NO and IL-10 [10–12]. However, it has been found that single LPS stimulation is insufficient for the upregulation of IL-12 production and there is a negative relationship between the consequences of extracellular derived calcium increase and the insufficient production of IL-12. For example, ligation of Fc*γ*, complement, or scavenger receptors in macrophage inhibited the induction of IL-12 by LPS. Moreover, further experimental approaches suggested that IL-12 inhibition was due to extracellular calcium influxes that occurred after receptor ligation [13]. In our previous study, we demonstrated that clathrin/dynamin-dependent internalization, a process that interplayed with calcium influx, also resulted in the elevation of IL-12 secretion in murine macrophages [14]. Such results suggest that calcium may activate different intracellular signaling events which may exert an opposite function for IL-12 regulation. However, the precise regulatory mechanisms require further elucidation.

Generally, intracellular calcium binds to calmodulin (CaM), activates CaM kinases, and further regulates transcriptional events in inflammation and immunity. Calcium/calmodulin dependent protein kinase kinase *β* (CaMKK*β*) is a key CaM kinase activated by the increased intracellular calcium and plays an essential role in calcium-mediated regulation of inflammation in innate immune cells [12]. AMP-activated protein kinase (AMPK) is a crucial substrate of CaMKK*β* which mainly functions as a cellular nutrient and energy sensor [15]. Recent studies reveal that AMPK is also an effective inhibitor for NF-*κ*B signaling and associated with inflammatory response in macrophages [12, 16]. Sirtuin 1 (SIRT1) is a key downstream regulator of AMPK in metabolism. Recently, SIRT1 has been identified to be required for AMPK-mediated inflammation inhibition [17]. As a NAD^+^-dependent deacetylating enzyme, SIRT1 could be activated by elevated NAD^+^ levels and then downregulates inflammation by directly deacetylating subunit of NF-*κ*B like p65 and Cel [18, 19]. In addition, SIRT1 could also reciprocally activate AMPK or induce phosphorylation of PCG-1*α*, which thereafter inhibits the RelA/p65-mediated NF-*κ*B signaling. Despite the well known effects of CaMKK*β*, AMPK, and SIRT1 on the modulation of inflammation, their concomitant involvement in mediating the regulation of calcium-dependent IL-12 production needs to be further studied.

In this study, we detected calcium influx across the plasma membrane triggered by LPS and observed its inhibitory effect on LPS induced IL-12 secretion in murine macrophages. We also evaluated the effects of CaMKK*β*-AMPK-SIRT1 signaling pathway on regulating NF-*κ*B activity and mediating calcium-dependent IL-12 inhibition.

## 2. Materials and Methods 

### 2.1. Cell Culture

Murine peritoneal macrophages were obtained from peritoneal lavage in BALB/c male mice as described previously [20]. Briefly, BALB/c mice were sacrificed and 1 mL high glucose DMEM (GIBCO, USA) was injected into the intraperitoneal cavity. The abdomen was gently massaged for 1 min and the injected medium was aspirated and cell pellets were washed twice with DMEM containing 10% fetal bovine serum (FBS). Then, peritoneal macrophages were plated in plates or dishes and cultured at 37°C in a humidified incubator supplemented with 5% CO_2_. Murine macrophage-like cell line RAW 264.7 cells (ATCC, USA) were directly cultured in DMEM supplemented with 10% FBS and under similar environment with primary macrophages.

### 2.2. Cell Treatment

For calcium modulation, macrophages were treated in calcium-free DMEM (GIBCO) or DMEM with EGTA (Sigma, USA) for calcium deprivation. Cells were treated with calcium-free DMEM plus 2 mM CaCl_2_ (Sigma) for calcium recovery. Then, macrophages were also treated with SKF96365, 2-aminoethyl diphenylborinate (2-APB) (Sigma), and LaCl_3_ (Aladdin, China) for inhibition of store-operated calcium entry (SOCE) or with ATP to increase calcium ATPase activity. Then, LPS O55: B5 (Sigma) was added and incubated for the indicated time periods before further measurement. For chemical modulation of CaMKK*β*, AMPK, SIRT1, NF-*κ*B, and AP-1, macrophages were pretreated with STO-609, AICAR, Compound C, wedelolactone (Sigma), SR11302 (Tocris, UK), and SRT1720 and EX527 (Selleck, USA) for 1 h and then stimulated with LPS before further measurement.

### 2.3. Intracellular Calcium Detection

Time-dependent intracellular calcium detection was performed with a Varioskan*™* Flash Multimode Reader (Thermo) using the fluorescent dye Fura-2AM (Sigma). Murine peritoneal macrophages were seeded in 96-well plates and preloaded with Fura-2AM for 60 min. Loaded cells were treated with or without LPS (1 *μ*g/mL). Online detection of Fura-2AM in live cells was performed every 10 s for 6 min via fluorescence excited at 340 and 380 nm and filtered at 510 nm. Ratios of 340/380 nm were calculated and compared.

### 2.4. siRNA Transient Transfection

CaMKK*β*, AMPK*α*, and SIRT1 were transiently knocked down by siRNA transfection following the manufacturer's instruction. Briefly, RAW 264.7 cells were cultured into 70% confluence. Negative control (NC) siRNA (Santa Cruz, USA) or siRNA for CaMKK*β*, AMPK*α* (Ruibo Biotech Ltd., China), and SIRT1 (Santa Cruz) were mixed with Lipofectamine 3000 reagent (Invitrogen, USA) and added to the medium. After 24 h of transfection, the culture medium was replaced and further treatment was performed.

### 2.5. Western Blot Analysis

RAW 264.7 cells were lysed with RIPA reagents (Pierce, Rockford, IL, USA) containing cocktail protease and phosphatase inhibitors (Roche, Switzerland). Plasma proteins were separated by SDS-PAGE and transferred onto PVDF membranes (Millipore, USA). Blots were blocked with 5% BSA for 1 h and incubated with primary antibodies (1 : 1000 dilutions) for CaMKK*β*, pCaMKK*β*, AMPK*α*, pAMPK*α*, SIRT1, I*κ*B*α*, IRF3, pIRF3, p38, p-p38, and tubulin (Cell Signaling, USA) at 4°C overnight. Then, the blots were further incubated with HRP-conjugated secondary IgG antibodies (1 : 2000 dilutions, Cell Signaling) at 37°C for 1 h. Chemiluminescence images were developed with SuperSignal Sensitivity Substrate Kit (Pierce) with a ChemiDoc XRS imaging system (Bio-Rad, USA).

### 2.6. Real-Time PCR

Total RNA extracted from RAW 264.7 cells by a TRIzol reagent (Roche) was reversely transcribed into cDNA with a ReverTra Ace-*α*-RNA easy Kit (Toyobo, Japan). The cDNA templates were mixed with SYBR Green PCR Master Mix (Toyobo) and PCR primers for IL-12 p35, IL-12 p40, TNF-*α*, IL-6, CaMKK*β*, AMPK*α*, and *β*-actin (sequences are listed in Table S1, in Supplementary Material available online at http://dx.doi.org/10.1155/2016/6152713). Quantitative real-time PCR was performed with an iCycler Thermal Cycler (Bio-Rad).

### 2.7. Luciferase Reporter Gene Assay

RAW 264.7 cells (1 × 10^5^/mL) were cultured in a 24-well plate and transfected with plasmids pGL-luc2P/NF-*κ*BRE (Promega, USA) and pAP1-luc and pIFN-*β*-luc (Genepharma, China) using X-tremeGENE HP DNA Transfection Reagent (Roche) for 24 h. Then, the plate was added with LPS for 6 h of incubation treatment. The luciferase activity was analyzed with the Firefly Luciferase Assay Kit (Beyotime) and detected in a multimode reader (Thermo). Relative luciferase light units were normalized to untreated cells.

### 2.8. ELISA Assay

Supernatants from cultured murine peritoneal macrophages or RAW 264.7 cells were directly collected after 24 h of LPS treatment or at a given time. The levels of IL-12 p40 and IL-12 p70 in the supernatant were measured with ELISA Kits (eBioscience, USA) as indicated by the manufacture's instruction.

### 2.9. NAD^+^/NADH Ratio Assay

RAW 264.7 cells (2 × 10^5^/mL) growing in 24-well plates were treated with LPS and LPS plus calcium-free DMEM and calcium-free DMEM with CaCl_2_ for 1 h. Cells were washed twice with PBS. NAD^+^/NADH levels in cell lyses were detected through a NADH Quantitative Detection Kit (Sigma). The procedures were performed as indicated by the manufacturer's instruction.

### 2.10. Statistical Analysis

Quantitative data were expressed as means ± standard deviation (SD). Student's *t*-test was used for comparisons between two groups. One-way ANOVA plus post hoc Bonferroni correction was used for multiple comparisons. Difference with a *P* value less than 0.05 and 0.01 was considered to be statistically significant.

## 3. Results

### 3.1. LPS Induces Secretion of TNF-*α*, IL-6, and IL-10 in Large Amounts but Stimulates a Much Lower Level of IL-12 Production in Murine Macrophages

**Figure 1:**
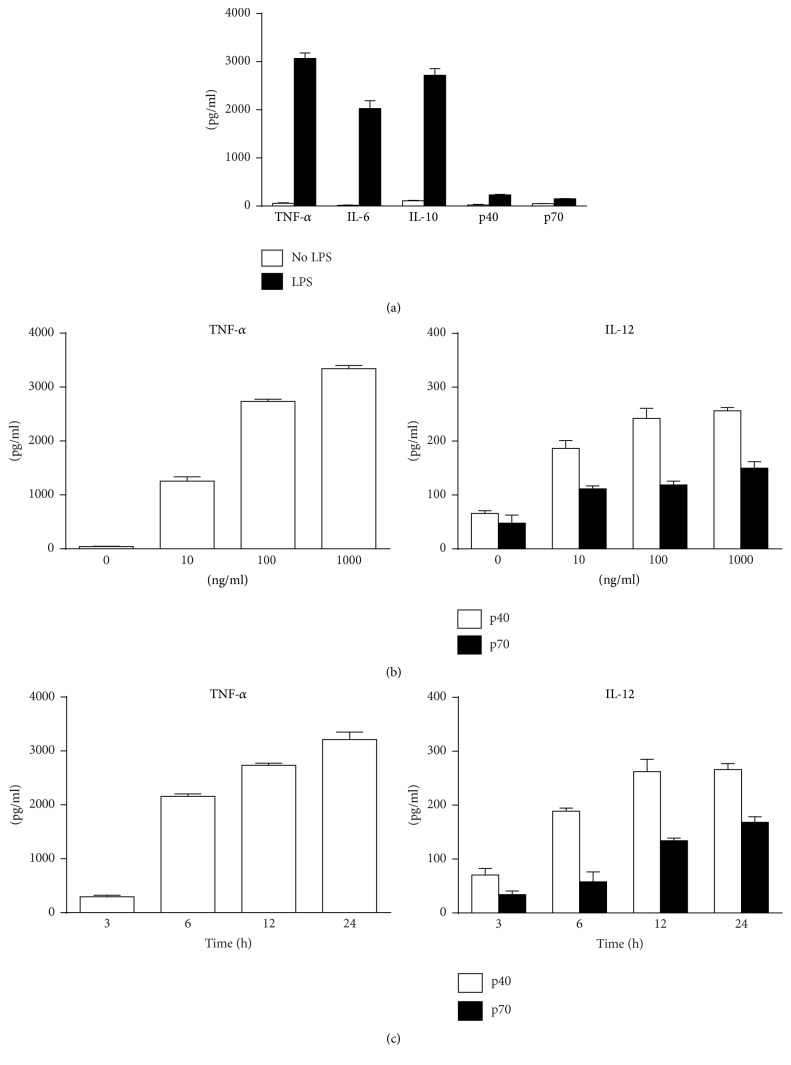
LPS induces the low levels of IL-12 in murine peritoneal macrophages. (a) Murine peritoneal macrophages were treated with or without 100 ng/mL LPS for 24 h. Supernatant levels of TNF-*α*, IL-6, IL-10, IL-12 p40, and IL-12 p70 were detected by ELISA. (b, c) Peritoneal macrophages were treated with LPS (0, 10, 100, and 1000 ng/mL) for 24 h (b) or with 100 ng/mL LPS for 3, 6, 12, and 24 h (c). Supernatant levels of TNF-*α* and IL-12 p40/p70 were detected by ELISA (*n* = 3).

To compare supernatant levels of TNF-*α*, IL-6, IL-10, and IL-12 (p40 and p70), primary murine peritoneal macrophages were treated with 100 ng/mL LPS. Results showed LPS induced dramatic increase of TNF-*α*, IL-6, and IL-10 secretion but triggered relatively lower concentration of IL-12 p40 and IL-12 p70 production (Figure 1(a)). Moreover, LPS (10, 100, and 1000 ng/mL) induced robust increase of TNF-*α* secretion in a significantly dose-dependent manner, but IL-12 secretion only increased slightly with elevated LPS concentration (Figure 1(b)). In the time course detection, the production of TNF-*α* increased rapidly 6 h after LPS stimulation. IL-12 p40 and IL-12 p70 were also elevated time dependently. However, their increases were much weaker (Figure 1(c)). Consistent trend was also detected in LPS-treated RAW 264.7 cells (Figure S1).

### 3.2. The Absence of Extracellular Calcium Selectively Upregulated IL-12 Expression in LPS-Treated Macrophages

**Figure 2:**
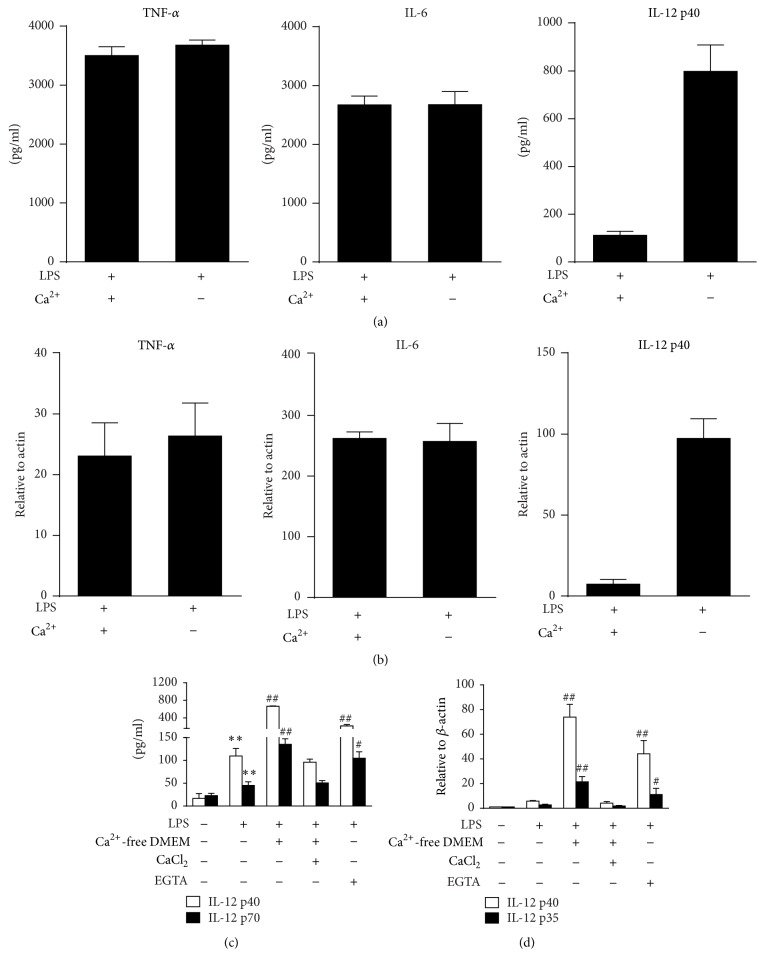
Depletion of extracellular calcium selectively upregulates IL-12 expression in LPS-treated murine macrophages. (a, b) Murine peritoneal macrophages cultured in DMEM with or without calcium for 1 h and then stimulated with LPS (100 ng/mL). Supernatants were collected after 24 h and the protein levels of TNF-*α*, IL-6, and IL-12 p40 were detected by ELISA ((a) *n* = 3). Total RNA was extracted after 12 h and mRNA expressions of TNF-*α*, IL-6, and IL-12 p40 were detected by real-time PCR ((b) *n* = 3). ^*∗∗*^ *P* < 0.01 versus LPS. (c, d) Murine peritoneal macrophages were incubated with LPS (100 ng/mL) in normal DMEM, calcium-free DMEM, calcium-free DMEM with 2 mM CaCl_2_, or normal DMEM with 5 mM EGTA. The mRNA expressions of IL-12 p40 and IL-12 p35 were detected via real-time PCR (c). The supernatant levels of IL-12 p40 and IL-12 p35 were detected via ELISA (d). ^*∗∗*^ *P* < 0.01 versus medium; ^#^ *P* < 0.05 and ^##^ *P* < 0.01 versus LPS (*n* = 3).

The influence of extracellular calcium on cytokines production was investigated by treating murine peritoneal macrophages in DMEM with or without calcium (calcium-free). The results showed there was no difference in the expression of TNF-*α* or IL-6 regardless of whether the medium contained calcium or not (Figures 2(a) and 2(b)). However, IL-12 p40 and IL-12 p70 were significantly upregulated without calcium. Similar changes of TNF-*α* and IL-12 p40 were detected in RAW 264.7 cells (Figure S2). Moreover, LPS induced production of IL-12 p40 was enhanced by cotreatment with IFN-*γ*. Deprivation of calcium further increased the IL-12 p40 production (Figure S3). The specificity of LPS induced IL-12 alteration due to calcium modulation was also identified by PMB cotreatment in RAW 264.7 cells. This specific LPS neutralizer effectively suppressed the elevated IL-12 p40 and IL-6 regardless of whether calcium is present or absent (Figure S4). To fully elucidate the influence of extracellular calcium on IL-12 production, murine peritoneal macrophages were treated in calcium-free DMEM with or without CaCl_2_ supplementation. Cells were also treated with EGTA, a calcium chelator. Calcium-free DMEM and EGTA consistently increased the secretion of IL-12 p40 and IL-12 p70 proteins. Accordingly, mRNA expressions of IL-12 p35 and IL-12 p40 were also significantly upregulated (Figures 2(c) and 2(d)). The elevated expression of IL-12 was terminated when CaCl_2_ was resupplied, suggesting that the inhibitory effect was calcium-dependent.

### 3.3. Depletion of Extracellular Calcium Dampens the Influx of Calcium and Thereby Inhibits IL-12 Expression in LPS-Stimulated Murine Macrophages

**Figure 3:**
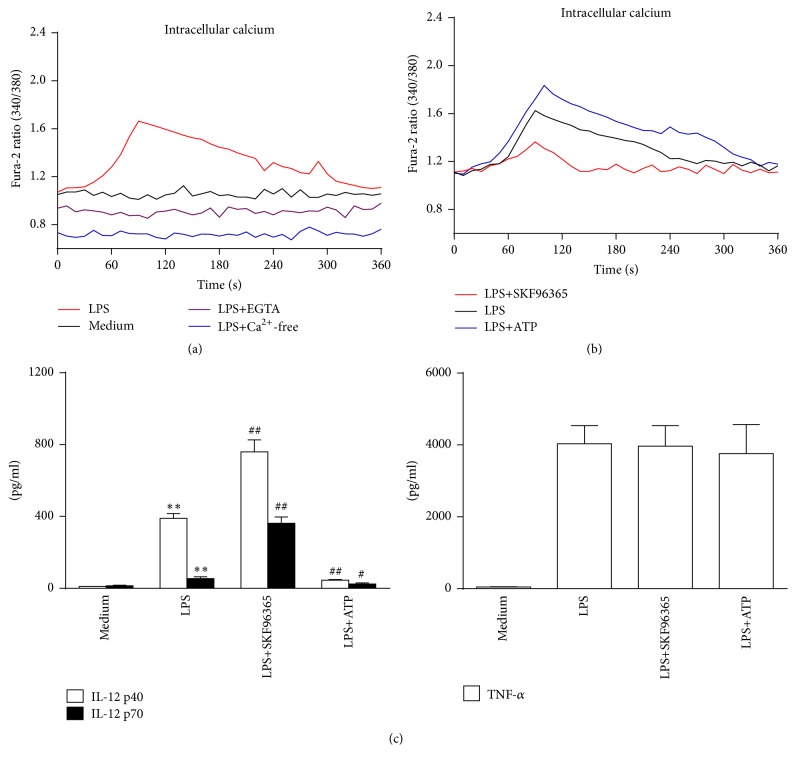
LPS induced extracellular calcium influx and modulation of calcium influx affects IL-12 production in murine peritoneal macrophages. (a, b) Macrophages were incubated with LPS (1 *μ*g/mL) or LPS plus calcium-free DMEM, 5 mM EGTA, 5 *μ*M SKF96365, or 100 *μ*M ATP for 6 min. The fluorescence ratio (340 nm/380 nm) of Fura-2AM was captured by using the plate reader assay every 10 s (*n* = 4). (c) Macrophages were treated with 5 *μ*M SKF96365 (SKF) or 100 *μ*M ATP for 1 h and then stimulated with 100 ng/mL LPS. Supernatants were collected 24 h later and IL-12 p40 and IL-12 p70 levels were detected by ELISA. ^*∗∗*^ *P* < 0.01 versus medium; ^#^ *P* < 0.05 versus LPS; ^##^ *P* < 0.01 versus LPS (*n* = 3).

Next, we detected the calcium influx in macrophages by LPS treatment. A transient elevation of intracellular calcium levels (indicated by Fura-2AM) was detected after LPS stimulation. The peak time came at 1-2 min after LPS treatment. Moreover, LPS induced increase of intracellular calcium levels was impaired when extracellular free calcium was removed (Figure 3(a)). These data suggest that transmembrane influx was induced by LPS which increased intracellular calcium levels. Besides direct extracellular calcium modulation, we further pretreated macrophages with SKF96365 and ATP, which either inhibited or activated the influx of calcium. In our study, SKF96365 treatment attenuated the influx of calcium and then resulted in the elevation of IL-12 p40 and IL-12 p70 production. In contrast, ATP treatment enhanced calcium influx and further decreased IL-12 secretion upon LPS stimulation. However, TNF-*α* release was not affected by SKF96365 or ATP (Figures 3(b) and 3(c)). We further detected the IL-12 modulating effects of other SOCE inhibitors (2-APB and LaCl_3_) and observed similar effects with SKF96365 (Figure S5). These results indicated that LPS induced intracellular calcium elevation by triggering extracellular calcium entry, which is also required for the negative regulation of IL-12 production in macrophages.

### 3.4. The Activation of CaMKK*β* Mediates Calcium-Dependent Inhibition of IL-12 Production in LPS-Activated Macrophages

**Figure 4:**
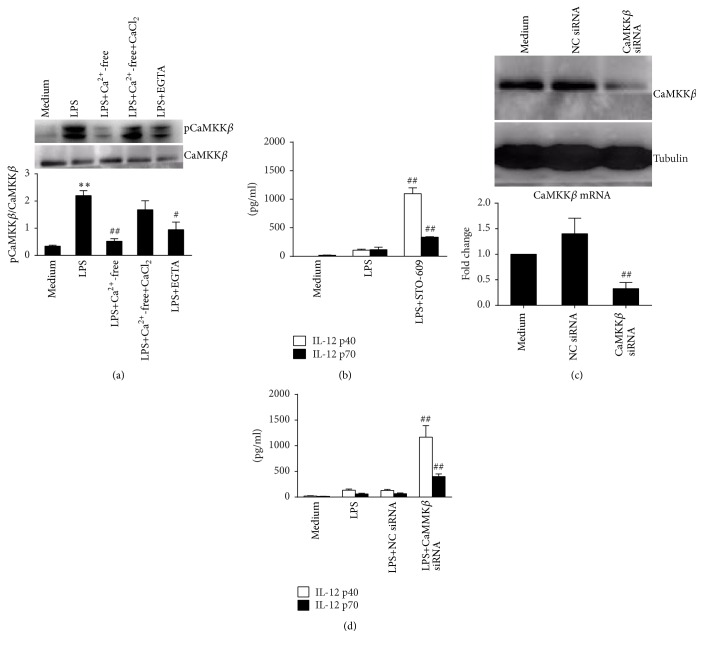
Activation of CaMKK*β* mediates the inhibition of calcium-dependent IL-2 production in RAW 264.7 cells. (a) Cells were treated with LPS in normal DMEM, calcium-free DMEM, calcium-free DMEM with 2 mM CaCl_2_, or DMEM with 5 mM EGTA for 30 min. Protein levels of CaMKK*β* and pCaMKK*β* were detected by western blot. Comparison was made for pCaMKK*β*/CaMKK*β*. ^*∗∗*^ *P* < 0.01 versus medium; ^#^ *P* < 0.05 and ^##^ *P* < 0.01 versus LPS (*n* = 3). (b) Cells were treated with LPS or LPS plus 1 *μ*M STO-609 for 24 h. Supernatants IL-12 p40 and IL-12 p70 were detected. ^##^ *P* < 0.01 versus LPS for each cytokine (*n* = 3). (c) Cells were treated with negative control siRNA (NC siRNA) or CaMKK*β* siRNA for 24 h. mRNA and protein levels of CaMKK*β* were detected by real-time PCR or western blot. ^##^ *P* < 0.01 versus NC siRNA (*n* = 3) for mRNA. (d) siRNA-treated cells were further stimulated by 100 ng/mL LPS for 24 h. Supernatants IL-12 p40 and IL-12 p70 were detected. ^##^ *P* < 0.01 versus LPS for each cytokine (*n* = 3).

To identify the downstream signaling effectors in calcium-mediated IL-12 inhibition, we detected CaMKK*β* activation in RAW 264.7 cells. In our study, LPS induced phosphorylation of CaMKK*β* was suppressed in medium without calcium or EGTA, both of which inhibited extracellular calcium influx. Restoration of CaMKK*β* phosphorylation was observed when extracellular calcium was resupplied (Figure 4(a)). To investigate whether activation of CaMKK*β* leads to IL-12 inhibition, we used chemical inhibitor STO-609 to suppress CaMKK*β* activation and found that STO-609 increased IL-12 production but did not affect the release of TNF-*α* induced by LPS (Figures 4(b) and S6). We further transfected specific siRNA to directly downregulate CaMKK*β* expression (Figure 4(c)). IL-12 production significantly increased in CaMKK*β* siRNA-treated groups (*P* < 0.01 versus LPS, Figure 4(d)). Such results demonstrate that the activation of CaMKK*β* is required for calcium associated IL-12 suppression in LPS-treated macrophages.

### 3.5. AMPK Is Activated Downstream of CaMKK*β* and Required for the Suppression of LPS Induced IL-12 Production in Macrophages

**Figure 5:**
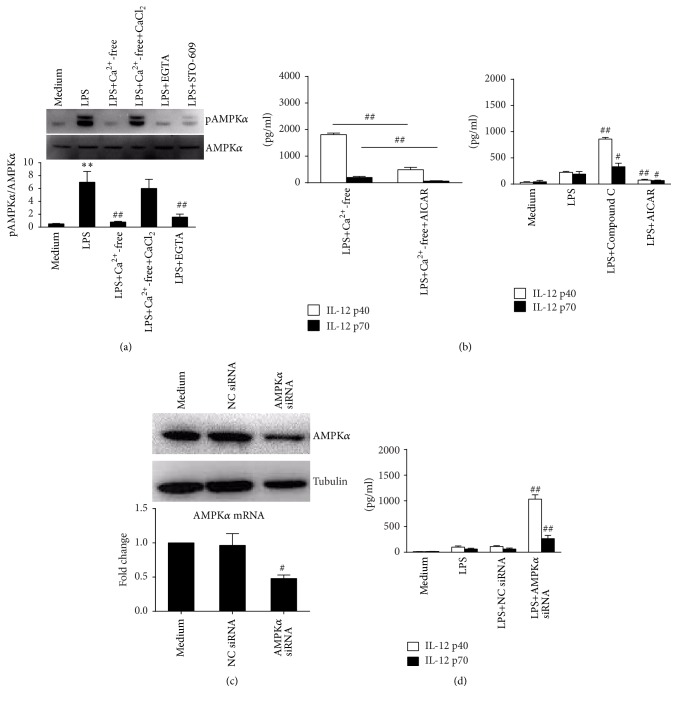
AMPK is activated downstream of CaMKK*β* for the negative control of the LPS induced IL-12 production in RAW 264.7 cells. (a) Cells were treated with LPS alone or with calcium-free DMEM, 2 mM CaCl_2_, 5 mM EGTA, or 1 *μ*M STO-609 for 30 min. Protein levels of AMPK*α* and pAMPK*α* were detected by western blot. ^*∗∗*^ *P* < 0.01 versus medium; ^#^ *P* < 0.05 or ^##^ *P* < 0.01 versus LPS (*n* = 3). (b) Cells were treated with LPS or LPS plus 1 mM AICAR in calcium-free DMEM (left) or with LPS, LPS plus 5 *μ*M Compound C, and LPS plus 1 mM AICAR in normal DMEM (right). Supernatants IL-12 p40 and IL-12 p70 were detected by ELISA. ^#^ *P* < 0.05 and ^##^ *P* < 0.01 versus LPS (*n* = 3). (c) Cells were treated with NC siRNA or AMPK*α* siRNA for 24 h. mRNA and protein levels of AMPK*α* were detected by real-time PCR or western blot. ^#^ *P* < 0.05 versus NC siRNA (*n* = 3) for mRNA. (d) siRNA-treated cells were further stimulated by 100 ng/mL LPS for 24 h. Supernatants IL-12 p40 and IL-12 p70 (24 h) were detected. ^#^ *P* < 0.05 and ^##^ *P* < 0.01 versus LPS for each cytokine (*n* = 3).

Next, we investigated whether AMPK was activated downstream of CaMKK*β* to mediate IL-12 inhibition. In our study, suppressed AMPK*α* phosphorylation (Thr 172) was detected in RAW 264.7 cells stimulated by LPS in calcium-free medium. AMPK phosphorylation was restored by supplementing CaCl_2_ recovery in the medium (Figure 5(a)). Moreover, EGTA and STO-609 also inhibited AMPK*α* phosphorylation. We further treated RAW 264.7 cells with AICAR (AMPK activator) or Compound C (AMPK inhibitor) before LPS stimulation. Whereas Compound C caused significant increase of IL-12 secretion, AICAR further decreased the production of IL-12 (Figure 5(b)). Then, addition of AICAR in calcium-free medium before LPS stimulation resulted in suppressed production of IL-12 p40 and IL-12 p70 (*P* < 0.01 versus LPS + Ca^2+^-free). We also transfected AMPK*α* siRNA in RAW 264.7 cells to directly decrease AMPK*α* expression (Figure 5(c)). Accordingly, the release of IL-12 p40 and IL-12 70 induced by LPS increased with AMPK*α* downregulation (*P* < 0.01 versus LPS, Figure 5(d)). These data collectively demonstrate that AMPK is activated downstream of CaMKK*β* and required for calcium-induced inhibition of IL-12 production in LPS-treated murine macrophages.

### 3.6. SIRT1 Is a Key Mediator Upregulated by AMPK Activation and Responsible for the Expression of IL-12 Expression

**Figure 6:**
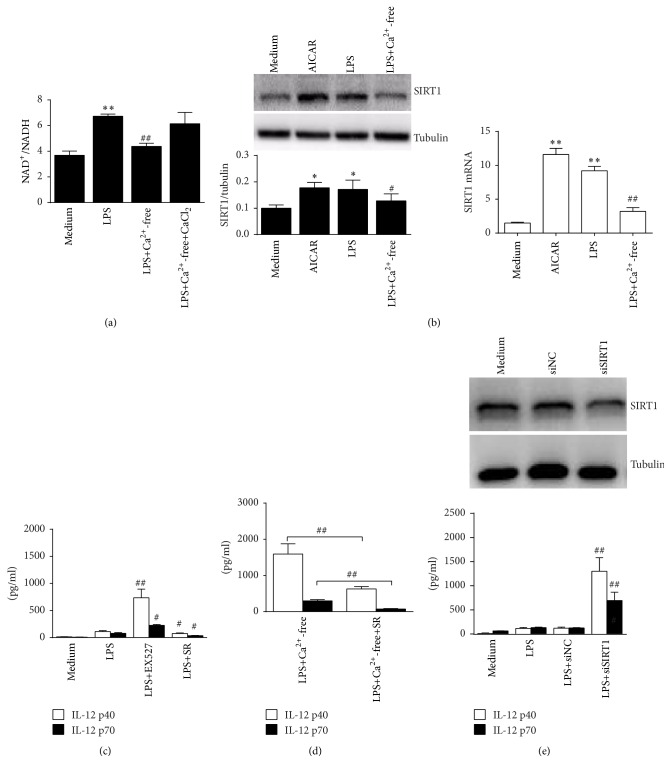
SIRT1 is upregulated by AMPK activation to suppress IL-12 expression in the LPS-activated RAW 264.7 cells. (a) Cells were treated with 100 ng/mL LPS in normal DMEM or in DMEM without calcium or supplemented with 2 mM CaCl_2_ for 1 h. NAD^+^/NADH levels were detected (*n* = 3). (b) Cells were treated with 1 mM AICAR, 100 ng/mL LPS, or LPS plus calcium-free DMEM for 2 h. Protein and mRNA levels of SIRT1 were detected by western blot or real-time PCR. ^*∗*^ *P* < 0.05 and ^*∗∗*^ *P* < 0.01 versus medium; ^#^ *P* < 0.05 or ^##^ *P* < 0.01 versus LPS (*n* = 3). (c, d) Cells were treated with LPS alone or with 2 *μ*M EXT527 or 2 *μ*M SRT1720 (c). Cells were also treated with LPS, calcium-free LPS, or calcium-free LPS with SRT1720 (d). Supernatants IL-12 p40 and IL-12 p70 (24 h) were detected by ELISA. ^#^ *P* < 0.05 and ^##^ *P* < 0.01 versus LPS for each cytokine (*n* = 3). (e) Cells were treated with NC siRNA or SIRT1 siRNA for 24 h. Protein levels of SIRT1 were detected by western blot (upper). The siRNA-treated cells were further stimulated by 100 ng/mL LPS for 24 h (lower). Supernatants IL-12 p40 and IL-12 p70 were detected. ^#^ *P* < 0.05 and ^##^ *P* < 0.01 versus LPS for each cytokine (*n* = 3).

SIRT1 is a substrate of AMPK in the regulation of inflammation, so we investigated whether SIRT1 was also required in mediating IL-12 inhibition. As SIRT1 activation is NAD^+^-dependent, we first detected the NAD^+^/NADH ratio in the presence or absence of extracellular calcium. In our study, LPS treatment alone induced elevated NAD^+^ levels, which was dampened by using calcium-free medium and restored by addition of CaCl_2_ (Figure 6(a)). Then, expressions of SIRT1 were upregulated by both LPS and AMPK activator AICAR in RAW 264.7 cells (Figure 6(b)). In accordance with NAD^+^ detection, LPS treatment in medium without calcium caused suppressed SIRT1 expression. Furthermore, the SIRT1 inhibitor, EX527, markedly increased the production of IL-12 p40 and IL-12 p70 while its agonist, SRT1720, suppressed the IL-12 secretion induced by LPS (Figure 6(c)). When SRT1720 was added in calcium-free medium before LPS stimulation, the enhanced IL-12 production was inhibited (Figure 6(d)). In addition, knockdown of SIRT1 by siRNA also markedly enhanced IL-12 production upon LPS stimulation in macrophages (Figure 6(e)). These data verify that SIRT1 may be essentially responsible for calcium-mediated IL-12 suppression.

### 3.7. SIRT1 Selectively Attenuates NF-*κ*B Activation Which Thereby Suppresses IL-12 Expression in LPS-Activated Macrophages

**Figure 7:**
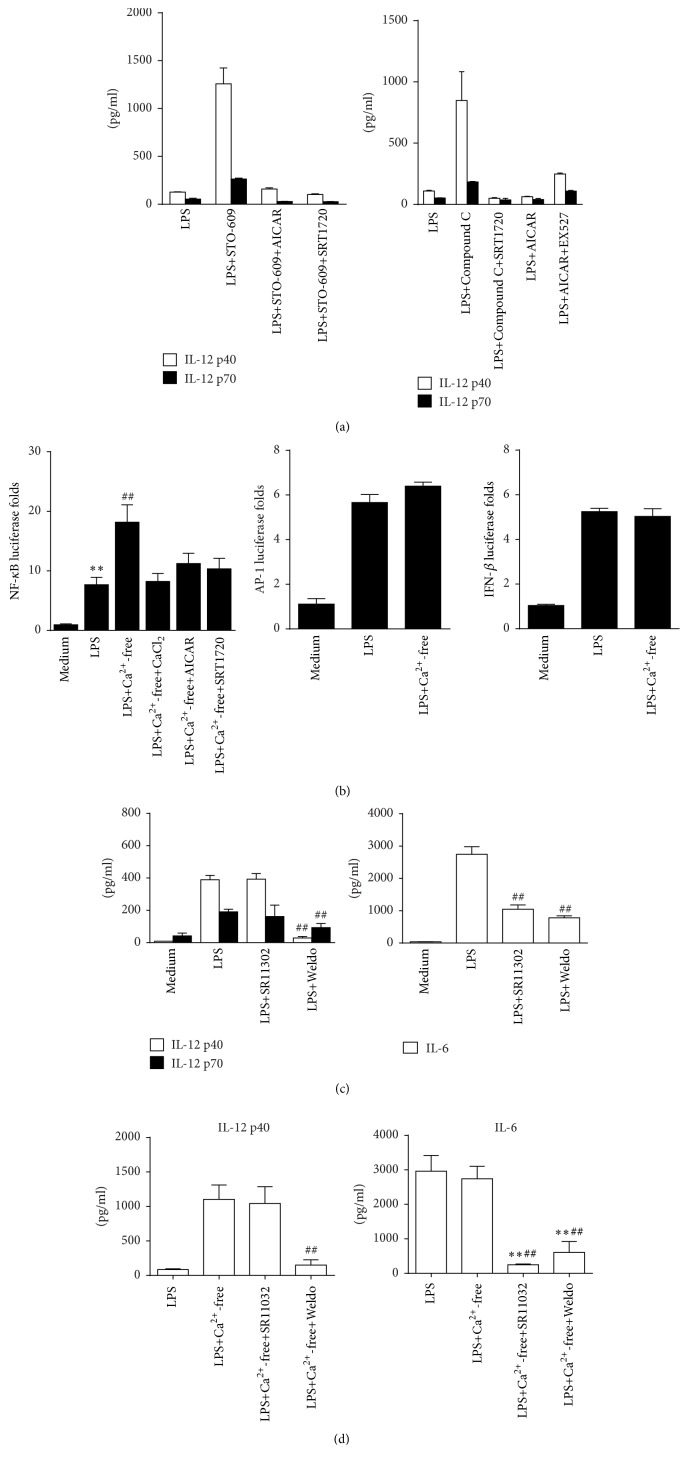
SIRT1 selectively attenuates transcription activity of NF-*κ*B and suppresses IL-12 expression in LPS-stimulated RAW 264.7 cells. (a) Cells were treated with 1 *μ*M STO-609 alone or combined with 2 *μ*M SRT1720 or 1 mM AICAR for 30 min before 100 ng/mL LPS stimulation (left). Cells were treated with 5 *μ*M Compound C alone or with 2 *μ*M SRT1720. Cells were also treated with 1 mM AICAR alone or with 2 *μ*M EX527 (right). Supernatants were collected at 24 h and IL-12 p40/IL-12 p70 (c) and IL-6 (d) levels were detected by ELISA (*n* = 3). (b) Cells were transfected with plasmid pGL-luc2P/NF-*κ*BRE for 24 h and further treated with 100 ng/mL LPS in normal DMEM, calcium-free DMEM, DMEM with 2 mM CaCl_2_, DMEM with 1 mM AICAR, or DMEM with 2 *μ*M SRT1720 for 6 h. Relative luciferase activity of NF-*κ*B against medium was presented. ^*∗∗*^ *P* < 0.01 versus medium; ^##^ *P* < 0.01 versus LPS (*n* = 3). Cells were also transfected with plasmids pAP1-luc and pIRF3-luc for 24 h and further treated with 100 ng/mL LPS and LPS plus calcium-free DMEM for 6 h. Relative luciferase activity of AP-1 and IRF3 was detected (*n* = 3). (c) Cells were treated with LPS alone with 5 *μ*M SR11302 or 10 *μ*M wedelolactone (Weldo) for 24 h. Supernatant IL-12 p40/p70 and IL-6 levels were detected by ELISA. ^##^ *P* < 0.01 versus LPS (*n* = 3). (d) Cells were treated with LPS, LPS with calcium-free medium, or LPS with calcium-free medium alone with 5 *μ*M SR11302 or 10 *μ*M wedelolactone (Weldo) for 24 h. Supernatant IL-12 p40/p70 and IL-6 levels were detected by ELISA. ^*∗∗*^ *P* < 0.01 versus LPS; ^##^ *P* < 0.01 versus LPS plus calcium-free medium (*n* = 3).

As calcium deprivation affects activation of CaMKK*β*, AMPK, and SIRT1, we next used combined modulator of these kinases to identify their connection in mediating IL-12 regulation. Our results demonstrated that AMPK and SIRT1 activation impaired the increased IL-12 production due to CaMKK*β* inhibition. Moreover, SIRT1 activation suppressed IL-12 released due to AMPK inhibition while SIRT1 inhibition partly restored IL-12 production suppressed by AMPK activation (Figure 7(a)).

SIRT1 suppresses inflammation driven by interfering with NF-*κ*B-activation, so we next detected whether NF-*κ*B pathway was specifically targeted by calcium and SIRT1 to downregulate IL-12 expression. In our study, the transcriptional activity of NF-*κ*B induced by LPS was enhanced in calcium-free medium. Cotreatment with CaCl_2_, AICAR, or SRT1720 in calcium-free medium, which restored calcium influx or activated AMPK and SIRT1, suppressed the elevated NF-*κ*B activity (Figure 7(a)). However, the absence of extracellular calcium did not affect the transcriptional activity of IRF3 and c-Jun (a subunit of AP-1) induced by LPS (Figure 7(b)). Moreover, a significant decrease of IL-12 production was detected by the cotreatment of NF-*κ*B inhibitor wedelolactone with LPS. However, AP-1 inhibitor, SR11302, did not alter IL-12 levels. In a parallel study, both wedelolactone and SR11302 significantly attenuated IL-6 production in RAW 264.7 cells (Figure 7(c)). Furthermore, we observed similar modulation on IL-12 p40 and IL-6 by SR11302 and wedelolactone in the absence of calcium (Figure 7(d)). These results suggest that LPS induced calcium influx mediates IL-12 inhibition by ultimate suppression of the NF-*κ*B signaling pathway.

## 4. Discussion

IL-12 is essentially involved in host immune response and increased evidences have demonstrated the critical effects of IL-12 deficiency that result in immunosuppression and increased susceptibility to infection [1, 21, 22]. However, the precise regulatory mechanisms of IL-12 expression remain unclear and thereby are worthy of investigation. In the present study, we report the finding of a calcium-dependent mechanism that may possibly explain the insufficient IL-12 expression in LPS-stimulated murine macrophages. This newly identified mechanism is verified to be based on observations that LPS stimulation induces extracellular calcium influx which activates CaMKK*β*, AMPK, and SIRT1, dampens NF-*κ*B activation, and eventually downregulated IL-12 production.

Macrophages are primary sources for the production of inflammatory cytokines when activated by pathogenic molecules such as LPS. However, levels of these cytokines can vary greatly due to the complicated and diversified regulatory mechanisms [23]. In our study, LPS-triggered production of IL-12 p40 and IL-12 p70 was significantly lower than those of TNF-*α*, IL-6, and IL-10 secreted in the same cell population of either primary or cell line of murine macrophages. However, the underlying mechanism was unclear. Previously, it was found that LPS could activate the calcium-dependent signaling and regulate cytokine production in innate and adaptive immune cells [24–26]. Additional studies provided more specific data that calcium might influence the production of IL-12 [12, 13]. In our study, the removal of extracellular calcium directly caused a selective suppression of IL-12 production upon LPS stimulation. Therefore, these data suggest calcium may act as an essential negative regulator for the production of IL-12.

Extracellular calcium flux in response to microbial stimuli has been demonstrated in both mouse macrophages and mouse DCs [27, 28]. More specifically, calcium influx mainly contributes to the increase of intracellular calcium in LPS-treated macrophages [11]. In our study, we consistently observed extracellular calcium influx induced by LPS treatment in murine peritoneal macrophages and also in RAW 264.7 cells (data not shown). Such a transmembrane transportation of calcium was then demonstrated to be associated with IL-12 regulation, as enhanced IL-12 production was detected in macrophages cultured in medium without calcium or with lack of free calcium (EGTA). It has been found that calcium influx is regulated by clathrin/dynamin-dependent endocytosis, calcium channel regulators, and calcium ATPases. For example, the dynamin inhibitor dynasore could block TRPV5/V6 activity and inhibit calcium influx in Jurkat cells [29]. We found previously that inhibition of endocytic pathway significantly suppressed LPS induced production of IL-12 p40 [14]. SOCE is a primary process for extracellular calcium influx across the plasma membrane. A number of direct channel blockers, including La^3+^, SKF96365, and 2-APB, were discovered and designed to inhibit SOCE [30, 31]. In contrast, plasma membrane Ca^2+^-ATPases use ATP to transport Ca^2+^. Therefore, ATP could enhance SOCE activity and facilitate calcium influx. The cytoplasmic calcium changes and calcium influx evoked by adenosine triphosphate (ATP) were widely observed in many different cell types [32]. Therefore, the SOCE based modulators are with opposite function in modulation calcium influx and thereby may facilitate the study of calcium-dependent regulation for IL-12.

CaMKK*β* is a key signaling molecule activated by increased intracellular calcium. In immune cells, CaMKK*β* expression is only found in monocyte/macrophage lineage and responsible for regulation of inflammation and immunity [14]. More recently, studies performed on myocytes and macrophages have reported that CaMKK*β* phosphorylation could be induced by calcium influx or LPS stimulation [33]. In our study, we found that phosphorylation of CaMKK*β* was induced by LPS stimulation and dependent on the increased calcium influx. To investigate the direct involvement of CaMKK*β* in regulation of IL-12 production, we used its specific chemical inhibitor (STO-609) and siRNA to interfere with CaMKK*β* activation and observed the marked increase of IL-12 release in LPS-stimulated murine macrophages [34]. The precise regulation patterns of CaMKK*β* on inflammation remain largely unclear. In some cases, it was demonstrated that CaMKK*β* was protective against neuroinflammation by activating AMPK [35]. However, other authors reported that CaMKK*β* was essentially involved in macrophage-mediated release of inflammatory cytokines [14]. Our finding demonstrated for the first time that CaMKK*β* activation selectively suppressed LPS induced IL-12 production in murine macrophages. Therefore, the nature of CaMKK*β* based regulation on inflammation and immunity still requires further study.

Whereas AMPK is a crucial sensor for energy balance, it has been also demonstrated that AMPK activation exerts significant anti-inflammatory and immunosuppressive effects on a variety of cell types. Recently, AMPK has been identified as a direct substrate of CaMKK*β* for inflammation modulation in macrophages and this is currently accepted molecular pattern that explains the anti-inflammatory efficacy of CaMKK*β* [36]. The enhanced AMPK*α* phosphorylation could be achieved by CaMKK*β* activation with or without LPS [35, 36]. However, we demonstrated that LPS-stimulated AMPK activation depends on calcium influx and CaMKK*β* activation. Moreover, AMPK activation was necessary for calcium-dependent inhibition of LPS induced IL-12 production. Firstly, AMPK activation by AICAR further inhibited the suppression of IL-12 production, while inhibition of AMPK by Compound C resulted in a significant elevation of IL-12 release [36]. In AMPK*α* knockdown macrophages, elevated IL-12 levels were also detected upon LPS stimulation. Secondly, the increase of IL-12 production due to calcium depletion was abrogated when AICAR was given simultaneously. So these results support our presumption that AMPK is activated by the influx of calcium and activation of CaMKK*β* to mediate IL-12 suppression.

The molecular mechanisms for AMPK are complicated. Emerging evidences demonstrated that AMPK can inhibit NF-*κ*B activation and suppress the expression of inflammatory genes. AMPK could also modulate its downstream targets including SIRT1, PGC-l*α*, p53, and FoxO3a, either directly or indirectly [37]. Recently, AMPK has been verified in coordination with another metabolic sensor, NAD^+^-dependent type III deacetylase SIRT1, to regulate energy metabolism and inflammation [18, 38]. AMPK regulates SIRT1 by increasing the NAD^+^/NADH ratio, which acts as a key upstream regulatory event that modulates the activity of SIRT1 and could be achieved by LPS induced TNF-*α* release in proinflammatory macrophages [38, 39]. SIRT1 expression could be modulated by LPS stimulation in macrophages/monocytes and upregulated SIRT1 expression was observed in resveratrol treated endothelial cells via the PRKA-AMPK pathway [40, 41]. Therefore, AMPK may act as an essential modulator for SIRT1 expression and we mainly focus on AMPK induced NAD^+^ elevation and SIRT1 activation in this study.

In a previous study, an enhancement of SIRT1 activity in response to zymosan stimuli was demonstrated to reduce transcriptional activation of IL-12 in dendritic cells [42]. In our study, we identified that LPS induced the increase of NAD^+^/NADH ratio in murine macrophages and upregulated the expression of SIRT1, both indicating the elevated activity of SIRT1. Similar trends were found after AMPK activation by AICAR. We also verified the requirement of calcium in facilitating LPS induced SIRT1 activation which was abolished in medium without calcium. This presumption was further supported by our findings that selective activation of SIRT1 (SRT1720) activity suppressed IL-12 production but selective inhibition of SIRT1 (EX527) increased IL-12 levels upon LPS treatment [43, 44]. Moreover, the increased IL-12 production due to calcium absence was abolished by SIRT1 activation.

To further clarify the regulatory patterns of CaMKKb-AMPK-SIRT1 signaling cascade, we used combined manipulation of agonist and antagonist for CaMKKbeta-AMPK and SIRT1. Results demonstrated that CaMKK*β* couples with AMPK and SIRT1 to mediate transcriptional inhibition on IL-12 production induced by LPS. To further establish the CaMKKb-AMPK-SIRT1 signaling cascade activation which further mediates the inhibition of NF-*κ*B activation by LPS, SIRT1 could negatively regulate inflammation by interfering with transcription events mediated by RelA/p65, PGC-1*α*, and PPAR*α* [18]. It has also been found that the activity of SIRT1 is closely related to NF-*κ*B complex in controlling the resolution of inflammation [17]. In our study, depletion of extracellular calcium facilitated activation of NF-*κ*B, which was abolished by the presence of CaCl_2_, AMPK activation (AICAR), or SIRT1 activation (SRT1720). In contrast, the activation of IRF3 or AP-1 was not affected in either group as compared with LPS alone. In our study, the I*κ*B-*α* based NF-*κ*B inhibitor wedelolactone [45] effectively attenuated IL-6 and IL-12 production in RAW 264.7 cells. The AP-1 inhibitor SR11302 [46] only attenuated IL-6 production while it did not affect IL-12 secretion, although there exist different interpretations for IL-12 production mediated by AP-1 as well as its upstream kinases such as ERK1/2, p38, and JNK. Our results indicated that NF-*κ*B may be more importantly required for IL-12 p40 production, especially in the case that calcium influx was inhibited. However, further study is still necessary to determine transcriptional details involved in the ultimate regulation of IL-12 transcription by calcium-CaMKK*β*-AMPK-SIRT1 pathway.

## 5. Conclusion

We demonstrate in this study that LPS induced production of IL-12 is affected by the increased intracellular calcium derived from extracellular influx. Such effects were mediated by activation of CaMKK*β*, AMPK*α*, and SIRT1 which ultimately lead to the suppression of NF-*κ*B and downregulation of IL-12 production. Depletion of calcium influx inhibits those signaling events and thus increases LPS induced IL-12 production. Our findings discover a new connection between calcium, cellular energy metabolism, and inflammation, which may provide therapeutic targets for the precise regulation of inflammatory disorders.

## Supplementary Material

The supplementary materials include one table and seven figures. In Table S1, Sequences of PCR primers was presented. Figure S1, S2 and S6 demonstrate similar effects on IL-12 production in RAW 264.7 cells as compared with murine primary peritoneal macrophages. Figure S3 describes effects of calcium deprivation on IL-12p40 production induced by LPS with or without IFN-γ. Figure S4 demonstrates that the LPS neutralizer PMB inhibited the upregulated production of IL-12 p40 and IL-6 in LPS treated murine peritoneal macrophages. Figure S5 describes that SOCE inhibitors 2-APB and LaCl3 selectively inhibited the upregulated production of IL-12p40 in LPS treated murine peritoneal macrophages. Figure S7 illustrates that the CaMKKβ inhibitor STO-609 does not affect TNF-α production in both RAW 264.7 cells and murine peritoneal macrophages.
