# Supplementary material for: Inhibition of Extracellular Calcium Influx Results in Enhanced IL-12 Production in LPS-Treated Murine Macrophages by Downregulation of the CaMKKβ-AMPK-SIRT1 Signaling Pathway
Source: Mediators Inflamm. 2016 May 30;2016:6152713. doi: 10.1155/2016/6152713 (PMC4904125; doi:10.1155/2016/6152713)
Supplement: Supplementary file 1 — The supplementary materials include one table and seven figures. In Table S1, Sequences of PCR primers was presented. Figure S1, S2 and S6 demonstrate similar effects on IL-12 production in RAW 264.7 cells as compared with murine primary peritoneal macrophages. Figure S3 describes effects of calcium deprivation on IL-12p40 production induced by LPS with or without IFN-γ. Figure S4 demonstrates that the LPS neutralizer PMB inhibited the upregulated production of IL-12 p40 and IL-6 in LPS treated murine peritoneal macrophages. Figure S5 describes that SOCE inhibitors 2-APB and LaCl3 selectively inhibited the upregulated production of IL-12p40 in LPS treated murine peritoneal macrophages. Figure S7 illustrates that the CaMKKβ inhibitor STO-609 does not affect TNF-α production in both RAW 264.7 cells and murine peritoneal macrophages. [file 6152713.f1.docx]

**Supplementary Materials**

**Table S1 Sequences of PCR primers**

| **Name** | **Primer Sequence** |
| --- | --- |
| β-actin | F: 5’-GGGAAATCGTGCGTGACATCAAAG-3’  R: 5’-CATACCCAAGAAGGAAGGCTGGAA-3’ |
| IL-12 p40 | F: 5’-AGGTGCGTTCCTCGTAGA GA-3’  R: 5’-AAAGCCAACCAAGCAGAAGA-3’ |
| IL-12 p35 | F: 5' CTGTGCCTTGGTAGCATCTA 3' |
|  | R: 5' TTTCACTCTGTAAGGGTCTG 3' |
| TNF-α | F: 5'-CAGGTTCTGTCCCTTTCACTCACT-3' |
|  | R: 5'-GTTCAGTAGACAGAAGAGCGTGGT-3' |
| IL-6 | F: 5′-TGGAGTACCATAGCTACCTGGAGT-3′ |
|  | R: 5′-TCCT-TAGCCACTCCTTCTGTGACT-3′ |
| IL-10 | F: 5′-CATCGATTTCTTCCCTGTGAA-3′ |
|  | R: 5′-TCTTGGAGCTTATTAAAGGCATTC-3′ |
| CAMKKβ | F:5’-CATGAATGG ACGCTGC-3’  R:5’-TGACAACGCCATAGGAGCC-3’ |
| AMPKα | F:5’-GTCGACG TAGCTCCAAGACC-3’  R: 5’-ATCGTTTTCCAGTCCCTGTG-3’ |

**Figure S1. LPS induces low levels of IL-12 in RAW 264.7 cells. (A**) Murine peritoneal macrophages or RAW 264.7 cells were treated with or without 100 ng/ml LPS for 24 h. Supernatant levels of TNF-α, IL-6, IL-10, IL-12 p40 (p40) and IL-12 p70 (p70) were detected by ELISA. (**B**) Cells were treated with LPS (0, 10, 100 and 1000 ng/ml) for 24 h. Supernatant levels of TNF-α and IL-12 p40/p70. (**C**) Cells were treated with 100 ng/ml LPS for 3, 6, 12 and 24 h. supernatant IL-12 p40 and p70 levels were detected by ELISA (n = 3).

**Figure S2. Depletion of extracellular calcium selectively upregulated IL-12 expression in LPS treated RAW 264.7 cells.** Cells preincubated in normal DMEM or calcium free DMEM for 1 h and then stimulated with LPS (100 ng/ml). Supernatants were collected 24 h after LPS stimulation and protein levels of TNF-α, IL-10 and IL-12 p40 were detected by ELISA (**A**, n = 3). Total RNA was extracted 12 h after LPS stimulation and mRNA expression of TNF-α, IL-10 and IL-12 p40 was detected by real-time PCR (**B**, n = 3).

**: p<0.01

**Figure S3. Effects of calcium deprivation on IL-12p40 production induced by LPS with or without IFN-γ. RAW 264.7 cells were treated with** normal DMEM or calcium free DMEM for 30 min and then stimulated by LPS (100 ng/ml) alone or together with IFN-γ (100 ng/ml). Supernatants were collected 24 h after LPS stimulation and protein levels of IL-12 p40 and IL-6 were detected by ELISA (n = 3). **: *P*<0.01

**Figure S4. PMB inhibited the upregulated production of IL-12p40 and IL-6 in LPS treated murine peritoneal macrophages.** Cells preincubated in normal DMEM or calcium free DMEM for 30 min and then stimulated with LPS (100 ng/ml) in the presence or absence of PMB (5 μM). Supernatants were collected 24 h after LPS stimulation and protein levels of IL-12 p40 and IL-6 were detected by ELISA (n = 4). **: *P*<0.01 *vs* LPS; #: *P*<0.05, ##: *P*<0.01 *vs* LPS+Ca^2+^ free.

**Figure S5. SOCE inhibitors 2-APB and LaCl3 selectively inhibited the upregulated production of IL-12p40 in LPS treated murine peritoneal macrophages.** Cells preincubated in 2APB (10 μM) or LaCl3 (10 μM) for 1 h and then stimulated with LPS (100 ng/ml). Supernatants were collected 24 h after LPS stimulation and protein levels of IL-12 p40/p70 and IL-6 were detected by ELISA (n = 3). **: *P*<0.01 *vs* medium; #: *P*<0.05, ##: *P*<0.01 *vs* LPS**.**

**Figyre S6. Effects of CaMKKβ inhibition on TNF-α productionin of murine peritoneal macrophages and RAW 264.7 cells.** Cells were treated with LPS or LPS plus 1 μM STO-609 for 24 h. Supernatant TNF-α were detected by ELISA
